# Renal Medullary Carcinoma: Case Report of an Aggressive Malignancy with Near-Complete Response to Dose-Dense Methotrexate, Vinblastine, Doxorubicin, and Cisplatin Chemotherapy

**DOI:** 10.1155/2014/615895

**Published:** 2014-08-19

**Authors:** Ali Imran Amjad, Hira Ali, Leonard J. Appleman, Jodi Maranchie, Stephen Jackman, Anil Parwani, Rajiv Dhir, Somak Roy, Rahul A. Parikh

**Affiliations:** ^1^Division of Hematology and Oncology, Department of Medicine, University of Pittsburgh Medical Center, Pittsburgh, PA 15232, USA; ^2^Division of General Internal Medicine, Department of Medicine, University of Pittsburgh Medical Center, Pittsburgh, PA 15232, USA; ^3^Department of Urology, University of Pittsburgh School of Medicine, Pittsburgh, PA 15232, USA; ^4^Department of Pathology, University of Pittsburgh Medical Center, Pittsburgh, PA 15232, USA

## Abstract

Renal medullary carcinoma (RMC) is a rare but aggressive malignancy affecting young individuals with sickle cell trait. Renal medullary carcinoma commonly presents with advanced or metastatic disease and is associated with a rapidly progressive clinical course and an extremely short overall survival measured in weeks to few months. Due to the rarity of RMC, there is no proven effective therapy and patients are often treated with platinum-based chemotherapy. We report near-complete radiological and pathological response in a patient treated with dose-dense MVAC (methotrexate, vinblastine, doxorubicin, and cisplatin) chemotherapy. The patient underwent consolidation nephrectomy and retroperitoneal lymph node dissection and had a 16-month progression-free survival, one of the longest reported in patients with RMC.

## 1. Background

Renal medullary carcinoma (RMC) was initially described in 1995 in a retrospective study of 34 patients collected over 22 years at the Armed Forces Institute of Pathology. Thirty-three of the 34 patients had sickle cell trait or sickle cell disease; the mean tumor size at diagnosis was 7 cm and median survival following surgery was 12 weeks (range 3–52 weeks) [1]. In a second smaller retrospective analysis with 6 patients, median age at diagnosis was 24.5 years and time from diagnosis to death was 3 months (range 1–7 months) [2]. Karyotyping was performed in 4 patients and revealed chromosome 11 monosomy in all 4 patients and chromosome 3 abnormalities in 2 patients [2]. A retrospective study performed in Brazil, a country with a high incidence of sickle cell trait, identified seven patients with mean age of 22 years (8–69 years) and tumor size ranging from 4 to 12 cm. In this retrospective analysis, survival of patients with advanced RMC ranged from 4 days to 9 months [3]. A number of other case reports and series have described the role of surgery, chemotherapy, immunotherapy, or radiation in the treatment of RMC and found rapid progression despite these therapies [3–6]. Rathmell and Monk initially reported the use of high-dose intensity MVAC chemotherapy in a case-series of three patients with RMC [7]. They noted a significant improvement in both palliation and survival with the use of this regimen. In the current paper, we describe a young 23-year-old subject with sickle cell trait, diagnosed with diffusely metastatic RMC. In spite of having advanced disease, she achieved an excellent response to aggressive cytotoxic chemotherapy with a progression-free survival (PFS) of 16 months.

## 2. Case Presentation

**Figure 1:**
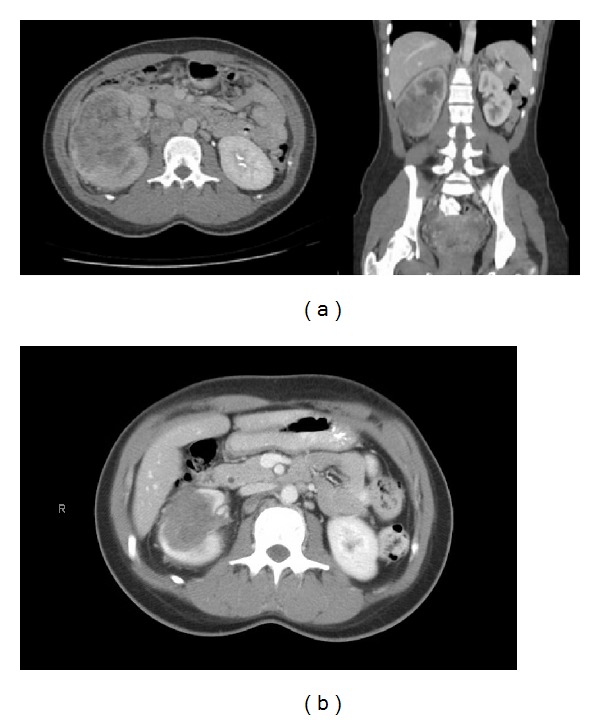
(a) CT scan of abdomen showing necrotic right-sided tumor replacing the kidney. (b) CT scan after chemotherapy.

A twenty-three-year-old African-American female with sickle cell trait presented to the emergency room with periumbilical and right-sided back pain associated with poor appetite and ten-pound weight loss over 4 months. She denied gross hematuria, dysuria, or additional urinary symptoms. She was a previously healthy nonsmoker with paternal family history of sickle cell trait. Physical examination was pertinent for fullness in the right flank and left supraclavicular lymphadenopathy. Laboratory findings revealed hemoglobin of 11.3 g/dL, platelet count of 269,000 per mm^3^, and normal LDH of 147 IU/L. Computed tomography (CT) scan of the neck, chest, abdomen, and pelvis demonstrated a 12 cm heterogeneous right renal mass (Figure 1(a)) with retrocaval, aortocaval, and paraaortic lymphadenopathy. In addition, there were bilateral pulmonary nodules (largest in right upper lobe measuring 1.8 × 1.4 cm), left pleural-based nodules (largest at the level of diaphragmatic pleura measuring 2.6 × 1.9 cm), and right hilar (2.5 × 2.2 cm), left supraclavicular, and bilateral cervical lymphadenopathy. MRI of the brain showed no evidence for intracranial metastasis.

**Figure 2:**
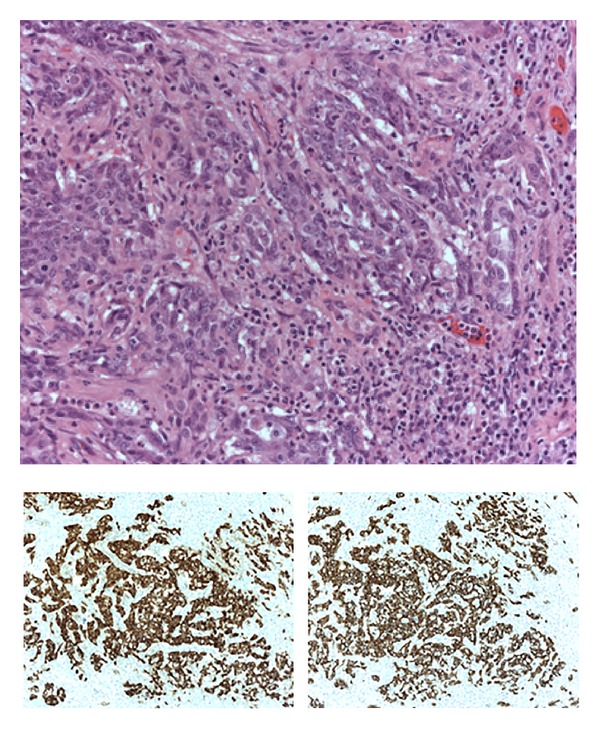
Supraclavicular lymph node (20x) with immunohistochemistry for CAM 5.2 and EMA.

Biopsy of left supraclavicular lymph node revealed high-grade renal medullary carcinoma with prominent lymphovascular tumor emboli. The tumor cells were eosinophilic, containing large nuclei and focally prominent nucleoli with brisk mitotic activity. The neoplastic cells stained positive for AE1/AE3, cytokeratin CAM 5.2, epithelial membrane antigen (EMA), E-cadherin, cytokeratin 7, and cytokeratin 19 on immunohistochemical analyses (Figure 2). The cells stained weakly positive for c-Kit and negative for cytokeratin 20, S100 protein, carbonic anhydrase IX, mucicarmine, and CD10. Cytogenetic studies performed on the lymph node isolated a clone of cells with duplication of the long arm of chromosome 1 (resulting in partial trisomy 1q) and a derivative chromosome 22 with chromatin material of unknown origin attached to the long arm (resulting in partial monosomy 22q). Fluorescence* in situ* hybridization (FISH) for transcription factor E3 (*TFE*) gene translocation was negative.

Treatment was initiated with dose-dense (dd) MVAC consisting of methotrexate 30 mg/m^2^ on day 1 followed by vinblastine 3 mg/m^2^, doxorubicin 30 mg/m^2^, and cisplatin 70 mg/m^2^ on day 2 of a 14-day cycle as described for metastatic urothelial carcinomas [8]. She received growth factor support with pegfilgastrim on day 3 to reduce the risk of neutropenic fevers. Treatment with four cycles of dd-MVAC was associated with a significant reduction in size of renal mass from 9.3 cm to 5.9 cm with areas of necrosis on a CT scan of chest, abdomen, and pelvis (Figure 1(b)). There was near-complete resolution of right upper lobe and left lower lobe parenchymal lung nodules and retroperitoneal lymphadenopathy. Multiple pleural-based nodules in the left hemithorax and right hilar lymph node decreased. Two additional cycles of dd-MVAC were administered and a follow-up CT scan showed continued improvement in size of renal mass to 4.4 cm, improvement in retroperitoneal, hilar lymphadenopathy, and parenchymal lung lesions. The subject developed grade I renal insufficiency and grade II chemotherapy-induced nausea and vomiting (CINV) for which the cisplatin dose was reduced by 25% after the initial two cycles. Overall, she tolerated six cycles of dd-MVAC extremely well, with no hospitalizations.

After achieving an excellent partial response to neoadjuvant chemotherapy, the patient underwent right radical nephrectomy with complete, bilateral retroperitoneal lymph node dissection without complications. Pathology revealed a gross tumor resection size of 6.2 cm. Viable carcinoma comprised approximately 2–4% of the tumor volume showing neoplastic cells with rhabdoid features in a background of extensive fibrosis and chronic interstitial inflammation. All surgical margins were negative for tumor. Two of 11 lymph nodes (one paracaval and one preaortic from the level of the aortic bifurcation) were involved with carcinoma (0.7 cm in greatest dimension) with no extracapsular extension. Pathologic TNM stage was assessed as pT1b N1 M1. Immunohistochemistry revealed a complete loss of SWI/SNF-related matrix-associated actin-dependent regulator of chromatin subfamily B member 1 (SMARCB1) expression. Subsequently, she underwent video assisted thoracoscopy and wedge resection of right lower lung lobe. Pathology showed inflammatory changes with no evidence of residual malignancy.

Follow-up imaging in the form of CT chest, abdomen, and pelvis was obtained every four months and at one year from diagnosis she showed no recurrence of disease. At 16 months from initial diagnosis, she developed shortness of breath and restaging scans showed disease recurrence with hepatic, pulmonary lesions and bilateral pleural effusions. She had a rapidly progressive decline after relapse and died without receiving any further therapy.

## 3. Discussion

Renal medullary carcinoma (RMC) is a rare form of aggressive kidney cancer (comprising <1% of all kidney cancers) and is exclusively restricted to patients with sickle cell trait or disease. It typically presents in young patients (male : female ratio 2 : 1) and follows an extremely aggressive clinical course with a poor outcome. We report a patient presenting with widely metastatic disease, with bulky renal primary and involvement of lung, pleura, and mediastinal and retroperitoneal lymph nodes. The patient had an excellent response to dose-dense MVAC regimen (methotrexate, vinblastine, doxorubicin, and cisplatin). After completion of six cycles of chemotherapy there was near-complete radiographic response and pathologic response at the primary 12 cm renal mass and surrounding lymph nodes. The patient eventually developed hepatic and pulmonary disease recurrence with a progression-free survival of 16 months.

Patients with RMC have an aggressive course and with cytotoxic regimens, survival is only weeks to a few months. Most patients respond to initial platinum-based chemotherapy but responses are rarely durable. The mechanism of this acquired resistance to cytotoxic chemotherapy is not clearly understood. The MVAC chemotherapy regimen, approved for use in advanced or metastatic transitional cell carcinoma of urinary bladder, has been previously utilized in RMC. Rathmell and Monk treated three patients with dose-dense MVAC and demonstrated partial responses with one patient achieving a 90% response by RECIST criteria after six cycles of chemotherapy. That patient relapsed and died at 12 months from initial diagnosis [7]. Walsh and colleagues reported a complete pathologic response to a combination of paclitaxel, gemcitabine with carboplatin in an 11-year-old patient with diffusely metastatic disease (lung, bone, and liver) [13]. This patient was diagnosed with left supraclavicular node biopsy and underwent neoadjuvant chemotherapy prior to resection of primary tumor and pulmonary metastasis. However, the patient had early relapse with leptomeningeal disease and died 24 months after initial diagnosis [13]. Very few other case reports of complete radiological responses have been described (Table 1). Pirich and colleagues initially reported a 12-year-old with sickle cell trait with a 6-month PFS to MVAC and an overall survival of 15 months [14].

A number of genetic abnormalities with potential pathophysiologic or therapeutic implications have been recently described in RMC. Translocation* t*(9; 22) involving bcr-abl has been described; however use of imatinib has not yielded any meaningful responses [9, 10]. Rearrangement of the* ALK* gene with translocation of the* ALK* receptor tyrosine kinase to a cytoskeleton protein vinculin has been described in a* t*(2; 10) bearing RMC [15]. In a recent study, RMC tumor specimens were noted to have an absence of SMARCB1/INI1 by immunohistochemistry. This correlates with loss of heterozygosity at the* SMARCB1/INI1* gene locus on chromosome 22 [16, 17]. Of note, our patient also demonstrated a loss of SMARCB1/INI1 expression by immunohistochemistry, with karyotyping revealing partial monosomy of chromosome 22q.* SMARCB1* is a tumor suppressor gene, which plays a role in chromatin remodeling, cell cycle control, and regulation of cytoskeletal dynamics [18–20]. It is not entirely clear how this gene may play a role in the pathogenesis of RMC. Prior reports have noted increased expression of hypoxia inducible factor (HIF), vascular endothelial growth factor (VEGF), and* TP53* in RMC [21]. Limited data on gene expression profiling of RMC using cDNA has shown resemblance to transitional cell carcinoma of the urinary bladder, which is supported with the rationale of treating these patients with MVAC, a regimen utilized commonly in transitional cell carcinomas of the bladder [22]. Whole-genome expression analysis in a patient who had complete remission for 9 months after second-line doxorubicin-based therapy showed high expression of topoisomerase II [23]. This expression profile may explain the unusual chemosensitivity of this patient to topoisomerase II inhibitor-based therapy. In the era of next generation sequencing, it is worth exploring whether sequencing this rare form of cancer will identify any driver-mutations, which can be targeted for therapeutic benefit.

## 4. Conclusion

In summary, we observed an excellent clinical and radiologic response in our patient with diffusely metastatic RMC with a PFS of 16 months on treatment with dd-MVAC followed by consolidation surgery. Thus, aggressive intervention of platinum-based chemotherapy can achieve prolonged responses with significant palliation of symptoms and should be strongly considered.

## Figures and Tables

**Table 1:** Case reports of complete radiological responses, regimens used, and survival of patients with renal medullary carcinoma.

Case report	Regimen described	Survival
Stahlschmidt et al. (1999) [9]	MVAC	11.2 months
Simpson et al. (2005) [10]	MVAC	11.2 months
Strouse et al. (2005) [11]	Cisplatin, gemcitabine, and paclitaxel	10 months and 11 months (*n* = 2)
Ronnen et al. (2006) [12]	Bortezomib	Alive with no evidence of disease at 27 months
Walsh et al. (2010) [13]	Carboplatin, gemcitabine, and paclitaxel	24 months

## Abbreviations

RMC: Renal medullary carcinoma
LDH: Lactate dehydrogenase
EMA: Epithelial membrane antigen
FISH: Fluorescence* in situ* hybridization
MVAC: Methotrexate, vinblastine, doxorubicin, and cisplatin
SMARCB1/INI-1: SWI/SNF related, matrix-associated, actin-dependent regulator of chromatin, subfamily b, member 1
RECIST: Response evaluation criteria in solid tumors.
